# Trends, Regional Variations, and Socioeconomic Disparities in Cesarean Births in India, 2010-2016

**DOI:** 10.1001/jamanetworkopen.2019.0526

**Published:** 2019-03-22

**Authors:** Christophe Z. Guilmoto, Alexandre Dumont

**Affiliations:** 1 Institut de Recherche Pour le Développement–Centre Population et Développement, Institut National de la Santé et de la Recherche Médicale, Université Paris Descartes, Paris, France

## Abstract

**Importance:**

The World Health Organization recommends that rates of cesarean delivery range from 10% to 15%. India has the largest annual number of births in the world and needs updates of existing estimates.

**Objective:**

To provide a new set of estimates of the rates of cesarean delivery and to map regional and socioeconomic disparities within these rates in India.

**Design, Setting, and Participants:**

Cross-sectional study primarily based on cross-sectional figures drawn from the fourth round of the National Family and Health Survey conducted from January 20, 2015, through December 4, 2016, by the Indian Institute for Population Sciences in Mumbai. The survey interviewed 699 686 girls and women aged 15 to 49 years and collected information on their last 3 pregnancies since January 2010 (259 627 births). The study population was statistically representative of India’s 36 states and Union territories and its 640 districts. The survey also included information on the socioeconomic status of households. The research is based on data tabulations and mapping and on spatial and regression analyses of microdata. Socioeconomic inequalities in access to cesarean deliveries were assessed using the Gini coefficient. Data were analyzed from August to October 2018.

**Main Outcomes and Measures:**

Rate of cesarean deliveries by regional and socioeconomic characteristics.

**Results:**

The cesarean birth rate computed for 699 686 Indian girls and women aged 15 to 49 years (mean [SD] age, 26.8 [5.0] years) was 17.2% (95% CI, 17.1%-17.3%) in 2010 to 2016, which corresponds to an estimated 4.38 million cesarean deliveries per year during the period (95% CI, 4.34-4.41 million) in India. Cesarean birth rates vary widely within the country, with a range of 5.8% (95% CI, 5.1%-6.5%) to 40.1% (95% CI, 38.4%-41.8%) across states and 4.4% (95% CI, 4.3%-4.6%) to 35.9% (35.4%-36.4%) across socioeconomic quintiles. The rate significantly increased from 9.2% (95% CI, 9.1%-9.3%) in 2004 to 2008. According to the recommended 10% to 15% benchmark of cesarean birth rates by the WHO, the estimated deficit of cesarean births in India is 0.5 million per year, whereas the estimated excess of cesarean births is 1.8 million. The overall Gini coefficient of inequality in access to cesarean deliveries is 46.4.

**Conclusions and Relevance:**

The rate of cesarean births is increasing in India and has already crossed the World Health Organization threshold of 15%. More research is needed to understand the factors behind the rapid rise of cesarean deliveries among affluent groups and in more developed regions.

## Key Points

**Question:**

What is the current proportion of cesarean births across India?

**Findings:**

In this cross-sectional study of 699 686 adolescent girls and women aged 15 to 49 years, the cesarean birth rate was 17.2% in 2010 through 2016, with variations ranging from 3% to 70% according to regions and socioeconomic groups.

**Meaning:**

India is characterized at the same time by a deficit of access to cesarean deliveries in poorer communities and the emerging overuse of cesarean delivery in more affluent regions and social groups.

## Teaser

This cross-sectional study estimates the rates of cesarean delivery and assesses regional and socioeconomic disparities within these rates among females of child-bearing age in India.

## Introduction

Cesarean deliveries are necessary surgical procedures in case of specific pregnancy and delivery complications. The use of cesarean deliveries has been increasing across the world during the last 2 decades.^1,2,3^ This rise is associated with significant reductions in maternal and neonatal mortality.^4,5^ Recent evidence also suggests that rates of cesarean delivery beyond a 15% threshold may lead to increased maternal and perinatal morbidity.^6,7^ This potential overuse of cesarean procedures places an additional burden on weak health systems in less developed countries with limited resources.

The slow growth of cesarean deliveries in India has been documented since the late 1980s.^8^ The first large-scale demographic surveys conducted in India estimated the cesarean rates at 2.9% (95% CI, 2.8%-3.0%) in 1988 to 1993 and at 7.1% (95% CI, 6.9%-7.3%) 6 years later (Table 1). During the following decade, the cesarean rate in India increased marginally to 8.5% (95% CI, 8.3%-8.7%) and 9.2% (95% CI, 9.1%-9.3%) according to the next 2 surveys conducted in 2005 to 2006 and 2007 to 2008, respectively. However, several hospital-based sources suggest that this rate increased faster during the last decade,^9,10^ a finding confirmed by the proportion of 17.2% (95% CI, 17.1%-17.3%) of cesarean births according to the fourth round of the National Family and Health Survey (NFHS-4) conducted from 2015 to 2016 for the reference period from 2010 through 2016.^17^

**Table 1.** Institutional Delivery and Cesarean Delivery Rates in India, 1988 to 2016

Survey	Period	Reference Period	Delivery Rate, % (95% CI)^a^			No. of Births
			Institutional	Population Cesarean	Institutional Cesarean	
NFHS-1	1992-1993	1988-1993	26.3 (25.9-26.7)	2.9 (2.8-3)	11.0 (10.8-11.3)	56 271
NFHS-2	1998-1999	1994-1999	34.0 (33.6-34.4)	7.1 (6.9-7.3)	20.9 (20.5-21.2)	53 179
DLHS-2	2002-2004	2000-2004	40.5 (40.3-40.7)	8.0 (7.8-8.1)	19.6 (19.5-19.8)	195 031
NFHS-3	2005-206	2001-2006	38.7 (38.3-39.1)	8.5 (8.3-8.7)	22.0 (21.6-22.3)	51 440
DLHS-3	2007-2008	2004-2008	46.9 (46.7-47.1)	9.2 (9.1-9.3)	19.0 (18.8-19.2)	217 997
NFHS-4	2015-2016	2010-2016	78.9 (78.7-79.1)	17.2 (17.1-17.3)	21.8 (21.6-21.9)	259 627

Abbreviations: DLHS, District-Level Household Survey; NFHS, National Health and Family Survey.

^a^ Computed from NFHS-4 microdata (version 73; released in May 2018).

Studies have described the trends and socioeconomic disparities in access to cesarean deliveries across the world,^11,12^ but the contribution of India to these worldwide trends remains largely undocumented, despite India’s importance. The annual number of births in India has been the largest in the world since the 1990s and will remain so during the 21st century.^13^ Using the latest population-based survey data, we herein describe the rapid increase in rates of cesarean deliveries in India and the extent of inequalities across regions and socioeconomic groups. We also examine whether our estimates confirm the presence of a negative gradient between cesarean rates and reproductive mortality described by previous studies.^4,5^

## Methods

### Sources to Study Cesarean Deliveries in India

In the absence of exhaustive civil registration and hospital figures in India, the proportion of cesarean births was estimated from the large-scale health surveys regularly conducted since the 1990s: the NFHS, the District-Level Household Surveys, and the Annual Health Survey.^14^ More recently, the Health Management Information System has provided annual rates of cesarean births, but the representativeness of these estimates remained untested.^15,16^ The figures used in the present analysis were drawn from these retrospective health and demographic surveys conducted in India during the last 20 years. The latest fourth round of the National Family and Health Survey (NFHS-4) was conducted from January 20, 2015, through December 4, 2016, by the Indian Institute for Population Sciences in Mumbai among a nationally representative sample of 601 509 Indian households. The NFHS-4 is based on a stratified sample with 28 586 primary sampling units, including 8397 urban units and 130 units classified as slums. The response rate was 97.6%.^17^ The procedure and questionnaire were reviewed and approved by the institutional review board. All respondents provided verbal informed consent with the interviewer as witness, who then completed the informed consent form on the interviewee’s behalf. This study followed the Strengthening the Reporting of Observational Studies in Epidemiology (STROBE) reporting guidelines.

The NFHS-4 figures were available for India’s 36 states and Union territories and 640 districts (2011 administrative boundaries). A total of 699 686 girls and women aged 15 to 49 years were interviewed during NFHS-4, with information collected on their last 3 pregnancies since January 2010 (259 627 live births). The mean sample size at district level was 406 live births (95% CI, 391-481), with 42 district units recording fewer than 200 live births during 2010 to 2016 and 162 with more than 500 live births. The information on cesarean procedures was available for the last 3 births since January 2010.

Cesarean delivery rates were assessed by asking mothers in 17 different languages the following question: “Was the child delivered by cesarean section, that is, did they cut your belly open to take the baby out?” The survey provided additional demographic and health information as well as household socioeconomic status computed by a principal component analysis based on housing type and household amenities.^18^ We used the latest NFHS-4 microdata sets (version 73; released in May 2018).

The quality of the NFHS-4 estimates of cesarean rates was tested against other existing figures, in particular from states where cesarean rates recorded an unusually fast increase between the last 2 NFHS rounds. This analysis showed the NFHS-4 figures to be consistent with prior estimates (eMethods 1, eTable 1, and eFigure 1 in the Supplement).

### Statistical Analysis

Data were analyzed from August to October 2018. We used the following software for individual- and district-level data: Stata (version 12; StataCorp) for statistical analysis, MapInfo (version 10; Pitney Bowes Software) for mapping, and GeoDa (version 1.2; Luc Anselin) for geostatistical analysis. The measure of spatial autocorrelation used was the Moran *I* index, ranging from 0 (no autocorrelation) to 1 (complete autocorrelation). This index measures the correlation coefficient between values observed in a district with the mean value of adjacent districts.

The cesarean rate was computed at the population level as the proportion of births reported in the survey delivered by cesarean delivery. The inequality within Indian states and between urban and rural India was assessed by cesarean rates computed over socioeconomic quintiles (eMethods 1 and eTable 2 in the Supplement). All results were weighted by sampling weights that corrected for sample design and are given with a 95% CI.

To assess the deficit and excess of cesarean births, we used the 10% to 15% threshold developed by the World Health Organization (WHO) in previous analyses. These cutoff points were only indicative. WHO stated that although the optimal rate is unknown, very low and very high rates of cesarean deliveries can reflect unmet needs or overuse, respectively.^1,7^ WHO also noted that the proposed upper limit (15%) should be seen as a threshold not to be exceeded: what matters most is the possibility that all the women have access to cesarean deliveries when they need them. Previous studies^4^ had shown that unmet needs corresponded to cesarean rates of less than 5% to 10%.

We considered proportions below or above this 10% to 15% range to represent a deficit or an excess of cesarean deliveries, respectively. To measure the extent of inequality in access to cesarean deliveries, we first divided India in 180 socioeconomic and regional subgroups (the mean number of births per subgroup is 1442) and applied the 10% to 15% threshold. We further used these 180 subgroups to rank deliveries by increasing rates of cesarean delivery to draw the Lorenz curve of concentration, which compares the observed distribution of cesarean deliveries with a hypothetical equal distribution (eFigure 2 in the Supplement). The Gini coefficient—ranging from 0 for perfect equal distribution to 100 for absolute inequality^19^—was computed as the proportion of the area between the line of perfect equality and the observed distribution. These computations are described in eMethods 2 in the Supplement.

## Results

### Trends and Regional Variations

The proportion of births delivered by cesarean delivery rose fast during the last decade in India. The cesarean rate computed on 699 686 Indian girls and women aged 15 to 49 years (mean [SD] age, 26.8 [5.0] years) reached 17.2% (95% CI, 17.1%-17.3%) in the period from 2010 to 2016 according to the NFHS-4 survey. The proportion doubled in 10 years (Table 1). The cesarean rates among institutional births (births taking place in health facilities) was slightly higher at 21.8% (95% CI, 21.6%-21.9%). Using figures of annual births in India during this period,^13^ we can estimate that 4.38 million births (95% CI, 4.34-4.41 million) were delivered by cesarean delivery every year during the period.

However, the national figure for cesarean births in a heterogeneous country such as India does not adequately reflect the diversity of the public health situations and challenges. Table 2 provides proportions of institutional and cesarean births in India’s largest states. Despite India’s rapid progress, Table 2 highlights that home deliveries still accounted for more than 30% of births in several populous states of North India such as Bihar and Uttar Pradesh. In contrast, institutional births represented more than 90% of births in all states of South India as well in other prosperous states such as Maharashtra and Punjab.

**Table 2.** Rates of Institutional Delivery and Cesarean Delivery by State and Union Territory^a^

State	Delivery Rate, % (95% CI)			No. of Births
	Institutional	Population Cesarean	Institutional Cesarean^b^	
Andhra Pradesh	91.5 (90.6-92.5)	40.1 (38.4-41.8)	43.8 (42.0-45.6)	3128
Arunachal Pradesh	52.3 (50.9-53.7)	8.9 (8.1-9.7)	17.0 (15.6-18.5)	4966
Assam	70.6 (69.8-71.5)	13.4 (12.8-14.1)	19.0 (18.1-19.9)	10 309
Bihar	63.8 (63.3-64.4)	6.2 (5.9-6.5)	9.7 (9.3-10.2)	25 437
Chhattisgarh	70.2 (69.3-71.2)	9.9 (9.3-10.5)	14.1 (13.2-14.9)	9283
Delhi	84.5 (82.7-86.3)	26.7 (24.6-28.9)	31.6 (29.2-34.1)	1580
Gujarat	88.7 (88.0-89.4)	18.4 (17.6-19.3)	20.7 (19.8-21.7)	7730
Haryana	80.4 (79.6-81.3)	11.7 (11.0-12.4)	14.5 (13.6-15.4)	7882
Himachal Pradesh	76.4 (74.9-78.0)	16.7 (15.3-18.0)	21.8 (20.1-23.5)	2929
Jammu and Kashmir	85.7 (84.9-86.4)	33.1 (32.1-34.1)	38.6 (37.5-39.8)	8245
Jharkhand	61.9 (61.1-62.8)	9.9 (9.4-10.4)	15.9 (15.1-16.8)	12 204
Karnataka	94.3 (93.8-94.8)	23.6 (22.7-24.6)	25.0 (24.0-26.0)	7789
Kerala	99.9 (99.8-100)	35.8 (33.9-37.7)	35.8 (33.9-37.7)	2462
Madhya Pradesh	80.8 (80.3-81.3)	8.6 (8.3-9.0)	10.7 (10.3-11.1)	24 611
Maharashtra	90.3 (89.7-90.9)	20.1 (19.3-20.9)	22.2 (21.4-23.1)	9401
Manipur	69.1 (67.9-70.3)	21.1 (20.1-22.2)	30.6 (29.1-32.1)	5636
Meghalaya	51.4 (49.9-52.9)	7.6 (6.8-8.4)	14.8 (13.3-16.2)	4409
Mizoram	79.8 (78.7-80.9)	12.7 (11.7-13.6)	15.9 (14.7-17.1)	4905
Nagaland	32.8 (31.5-34.2)	5.8 (5.1-6.5)	17.6 (15.7-19.6)	4607
Odisha	85.4 (84.8-86.1)	13.8 (13.2-14.5)	16.1 (15.4-16.9)	11 106
Puducherry	99.9 (99.7-100)	33.6 (30.8-36.4)	33.6 (30.8-36.4)	1081
Punjab	90.5 (89.7-91.3)	24.6 (23.4-25.7)	27.2 (25.9-28.4)	5216
Rajasthan	84.0 (83.4-84.5)	8.6 (8.2-9.0)	10.2 (9.7-10.7)	16 832
Sikkim	94.7 (93.3-96.1)	20.9 (18.4-23.4)	22.0 (19.4-24.7)	1005
Tamil Nadu	98.9 (98.7-99.2)	34.1 (33.0-35.1)	34.4 (33.4-35.5)	7922
Telangana	91.7 (90.6-92.8)	57.7 (55.8-59.7)	62.9 (60.9-64.9)	2427
Tripura	79.9 (77.7-82.0)	20.5 (18.3-22.6)	25.6 (23.0-28.3)	1330
Uttar Pradesh	67.8 (67.4-68.3)	9.4 (9.1-9.7)	13.8 (13.4-14.2)	41 751
Uttarakhand	68.6 (67.5-69.8)	13.1 (12.3-14.0)	19.1 (17.9-20.3)	5825
West Bengal	75.2 (74.0-76.4)	23.8 (22.7-25.0)	31.7 (30.2-33.1)	5328
**India**	**78.9 (78.8-79.1)**	**17.2 (17.1-17.3)**	**21.8 (21.6-21.9)**	**259 627**

^a^ Data are from the fourth round of the 2015-2016 National Family Health Survey (NFHS-4) and computed from NFHS-4 microdata set (version 73; released in May 2018). States with fewer than 1000 sample births are not shown.

^b^ Computed for births in health facilities.

Variations in cesarean deliveries were even wider: the cesarean rate varied from less than 10% in less developed states of North-Central India—from Rajasthan to Bihar and Madhya Pradesh—to values well above the WHO threshold of 15% in South India and in other more prosperous states. When restricted to institutional births, variations in cesarean rates remained pronounced across regions, with institutional cesarean rates close to 10% in North Indian states compared with 30% to 60% in more developed regions such as Delhi or South India.

**Figure 1.**
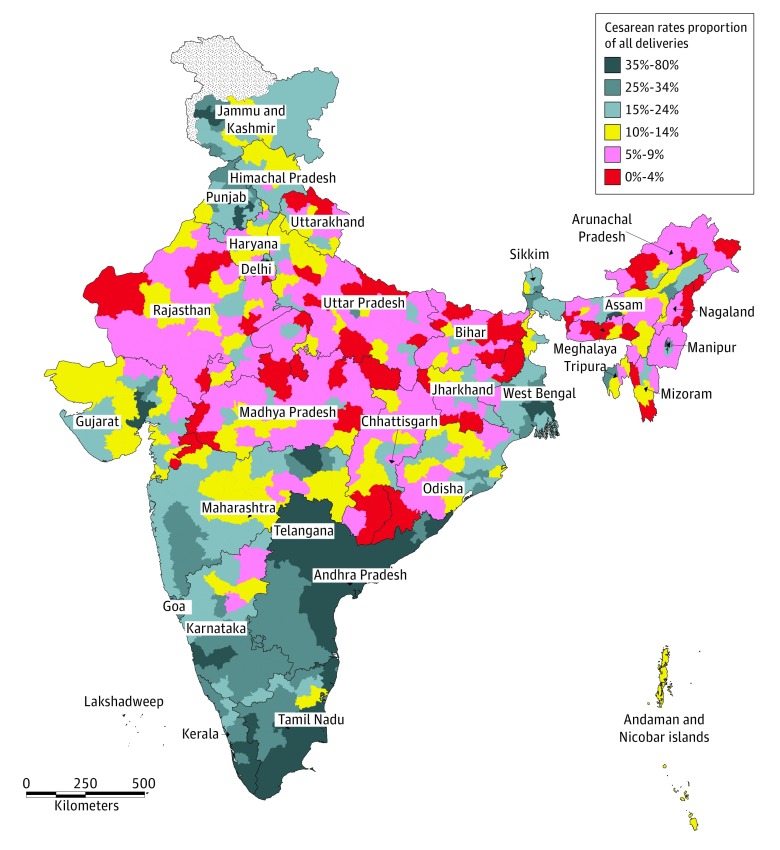
Proportions of Cesarean Deliveries in India by District Data are from the fourth round of the 2015-2016 National Family Health Survey (NFHS-4). The proportion of cesarean deliveries was computed for all births registered by the NFHS-4 survey in the 640 districts of India (administrative divisions at the time of the 2011 census). Values below the World Health Organization threshold of 10% to 15% threshold are shown in red; those above the threshold, in blue.

The district level map (Figure 1) provided a more systematic picture of regional differentials. Cesarean rates for India’s 640 districts illustrated the stark contrasts across regions. We first identified a large stretch of North India extending from Rajasthan to Bihar and Jharkhand where less than 10% of births were cesarean deliveries. This area included large patches of mountainous or forested districts of Northeast India, Uttarakhand, South Chhattisgarh, and Southwest Odisha. This broad territory clearly corresponded to the least developed areas of India—the Empowered Action Group states—but also included many northeastern states such as Nagaland, Meghalaya, or Arunachal Pradesh.

The highest cesarean rates, on the contrary, were observed in South India, notably in Telangana, Andhra Pradesh, and Kerala, as well as in most of Karnataka and Tamil Nadu, where cesarean rates were often greater than 35%. In 21 districts, more than half of the births were delivered by cesarean delivery. The apparent geographic concentration of cesarean rates illustrated by the map was further confirmed by a strong Moran index of spatial autocorrelation (*I* = 0.698).

### Socioeconomic Differentials

The analysis of data by wealth quintile demonstrated the extent of socioeconomic variations existing in India (eTable 2 in the Supplement). The cesarean rate increased from 4.4% (95% CI, 4.3%-4.6%) among the poorest quintile to 35.9% (95% CI, 35.4%-36.4%) among the richest. Similar variations across socioeconomic groups existed in rural and urban areas, although cesarean rates were higher in urban areas.

**Figure 2.**
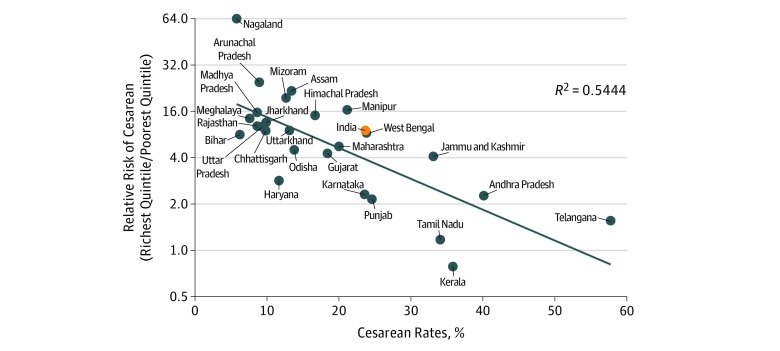
Cesarean Rates and Relative Risk by Socioeconomic Quintile for Larger States Data are from the fourth round of the 2015-2016 National Family Health Survey (NFHS-4). Cesarean rates at state level were compared with the relative risk ratio of cesarean delivery (calculated as the rates among the richest quintile divided by the rates among the poorest quintile). This relative risk ratio measures inequality in access to cesarean deliveries for the larger states. Confidence intervals are given in eTable 2 in the Supplement. Diagonal line indicates regression.

Among the 2 poorest quintiles accounting for 40% of population, the proportion of cesarean deliveries in India was below the 10% threshold in almost all Indian states—with the exception of the 5 South Indian states (Andhra Pradesh, Karnataka, Kerala, Tamil Nadu, and Telangana). Rates below 5% among the poorest quintile were also observed in the less developed states of North India (Bihar, Madhya Pradesh, Rajasthan, and Uttar Pradesh), which recorded more than 40% of all Indian births. Figure 2 compares the mean cesarean rates at state level with the interquintile ratios. This analysis demonstrated that inequality in the proportion of cesarean deliveries such as Rajasthan (interquintile ratio, 9.8) or Uttar Pradesh (interquintile ratio, 9.7). In contrast, the interquintile ratio declined with more frequent use of cesarean procedures, and interquintile disparities partly disappeared among South Indian states such as Kerala (interquintile ratio, 0.8) and Tamil Nadu (interquintile ratio, 1.4).

The true extent of inequalities in access to cesarean deliveries within India was best summarized by the Lorenz concentration curve (eFigure 2 in the Supplement). The curve illustrated the gap between Indian subpopulations classified by regional and socioeconomic variations: the less privileged 40% of births accounted only for 10% of all cesarean deliveries, whereas less than 20% of the births among better-off populations represented half of the cesarean deliveries. The resulting Gini coefficient of inequality was 46.4, which means that 46.4% of all observed cesarean births should take place among more disadvantaged groups for the distribution of cesarean deliveries to be equitable.

### Deficit and Excess of Cesarean Births in India

The cesarean rate in India in the period from 2010 to 2016 at 17.2% was only slightly above 15% limit proposed by the WHO. However, these figures did not correspond to a mere 2.2% excess above the 15% threshold. On the one hand, no less than 21.0% of deliveries still took place at home, among populations completely deprived of access to cesarean deliveries for economic or accessibility reasons. On the other hand, 27.1% of births were delivered in private clinics where the cesarean rate reached a record level of 40.4%, well above the WHO threshold.

The estimation of the deficit and excess of cesarean deliveries requires therefore a disaggregation of the Indian sample into more homogenous subgroups based on socioeconomic status and regional characteristics (eMethods 2 in the Supplement). This analysis indicates that 97 of 180 subgroups registered cesarean rates above the 15% benchmark. When cumulated, these births among more privileged populations correspond to an overall excess of cesarean deliveries for 7.0% of all deliveries. In contrast, a substantial number of subgroups (65 of 180) recorded rates below 10%. The overall shortfall in cesarean births among them represents 2.2% of deliveries in India.

Based on this disaggregated analysis, we can estimate that India recorded an annual excess of 1.8 million cesarean births from 2010 through 2016, concentrated in more advanced regions and affluent classes. During the same period, India recorded an annual deficit of 0.5 million cesarean births concentrated in underprivileged regions and populations among whom home deliveries remained frequent and cesarean rates remained low even among births in health facilities (Table 2).

### Cesarean Rates and Mortality Outcomes

**Figure 3.**
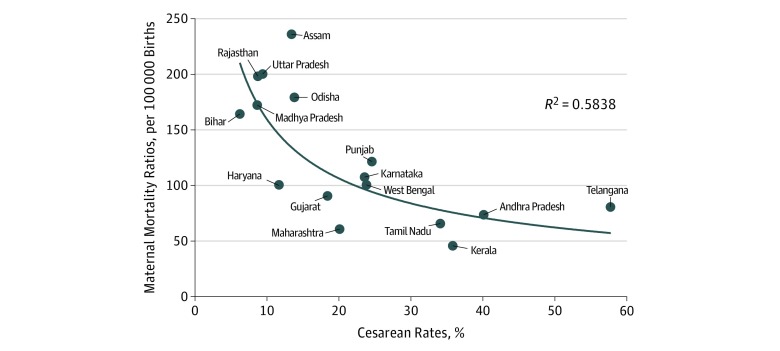
Cesarean Rates and Maternal Mortality Ratios in India for Larger States Data are for cesarean rates are from the fourth round of the 2015-2016 National Family Health Survey (NFHS-4); maternity mortality rates, 2014-2016 Sample Registration System estimation.^20^ Cesarean rates at state level were compared with the maternal mortality (calculated as the number of female deaths due to any cause related to or aggravated by pregnancy) ratio.

The benefits of access to cesarean deliveries can be highlighted by comparing delivery figures at state level with reproductive mortality indicators. In Figure 3 and eFigure 3 in the Supplement, we plotted cesarean rates against maternal mortality and neonatal mortality, respectively. The neonatal mortality estimates for states with more than 20 million inhabitants related to the 5 years preceding the NFHS-4 survey. Maternal mortality estimates from the Sample Registration System were available only for 16 states and related to 2014 to 2016.^20^

Figure 3 and eFigure 3 in the Supplement illustrate the strong negative relationship existing between cesarean and mortality rates at state level (significant at the 5% level). The lowest access rates to cesarean deliveries (<10%) were associated with maternal mortality ratios above 165 per 100 000 births, distinctly higher than the national ratios (130 per 100 000 births). Similarly, these low cesarean rates corresponded to neonatal mortality levels above 36 per 1000 births, well above the national mean (29.5 per 1000 births). At rates above 20%, the fitted curves tend to flatten out. The states with the highest cesarean rates (Andhra Pradesh and Telangana) did not register the lowest maternal or neonatal mortality rates.

## Discussion

The doubling in cesarean deliveries during the last decade is closely related to the simultaneous decline in the share of home deliveries without medical supervision. Their proportion decreased from 53.1% to 61.3% during the last 2 surveys in 2005 to 2008 (NFHS-3 and District-Level Household Survey) to 21.1% in 2010 to 2016. Since the mid-2000s, India has witnessed a revolution in access to modern delivery facilities, spearheaded by the Janani Suraksha Yojana program introduced in 2005^21^ and supplemented in 2011 with the Janani Shishu Suraksha Karyakram program.^22^ These new policies offer in particular conditional cash transfers to encourage women to use free prenatal and postnatal care in modern health centers, with additional cash benefits for health workers in charge of the coordination in more vulnerable states.^23,24^ The programs explain to a large extent the doubling of the proportion of institutional deliveries during the last decade (Table 1).

The role of public facilities in this revolution was crucial because 65.9% of all institutional deliveries occurred in government hospitals in 2015 to 2016, compared with 47% 10 years earlier (NFHS-3). However, the cesarean birth rates remained appropriate in government facilities at 11.9% in 2015 to 2016. In contrast, the cesarean rate in private clinics grew from 27.5% to 40.8% during the same period. Therefore, the last decade witnessed a complex transition affecting places and modes of delivery: many poor women gained access for the first time to safer childbirth in public facilities, while middle-class women delivered in larger numbers in private clinics, where the frequency of cesarean deliveries recorded a rapid increase.

Our analysis further shows that the diffusion of cesarean deliveries appears closely associated with socioeconomic status, with a variation in cesarean rates from 4.4% (95% CI, 4.3%-4.6%) to 35.9% (95% CI, 35.4%-36.4%) as women move up the social ladder (eTable 2 in the Supplement). The resulting interquintile ratio of 8.0 (95% CI, 7.8-8.4) is much higher in India than in other low- and middle-income countries.^11^ In addition, the geographic analysis of the 640 districts underscores the presence of considerable disparities across regions. Our demographic estimates highlight the persisting deficit in cesarean deliveries in India (half a million per year during the study period), a situation mostly attributable to the incidence of home deliveries among the poor and in less advanced regions. The statistical analysis points to the link of cesarean rates with lower levels of maternal and neonatal mortality. As in other parts of the world, the mortality benefits of increased cesarean deliveries are considerable at a low level of access to cesarean deliveries, but they tend to level out at higher cesarean rates with increased associated maternity morbidity.^4,5,7,25^

India’s Health Management Information System^26^ reports a steady increase in institutional deliveries from 70.6% in 2008 to 2009 to 92.3% in 2017 to 2018. At this rate, almost all Indian births are anticipated to take place in health facilities by the early 2020s, with access to surgical deliveries granted to most pregnant women. This source^26^ also reports a concurrent doubling of cesarean rates from 9.0% in 2008 to 2009 to 18.7% in 2017 to 2018 among institutional births. The proportion of cesarean deliveries should therefore continue to progress in the future well above the 15% threshold as a result of better access to health facilities, rising prosperity, lower fertility, and growing investment in pregnancies and childbirth. The upper limit of cesarean rates in India is suggested by levels already observed in private clinics (40.4%), among the richest quintile (36.0%), or in some South Indian states (35%-40%). These figures may also be compared with rates in 2014 estimated in emerging Asian countries such as China (34.9%) or South Korea (39.1%).^27^

The current cesarean rate corresponds to an estimated 4.38 million births per year (95% CI, 4.34-4.41 million) delivered by cesarean delivery in 2010 to 2016. The change during the last decade corresponds to an annual rate of increase in cesarean deliveries in India of 7%, almost twice the rate observed in the world.^1^ The total Indian figure is still below the 5.3 million cesarean births estimated in China in 2008 to 2014.^28^ However, the number of annual births in China is decreasing much faster than in India, and the cesarean rate is reportedly contracting.^29^ Therefore, in a matter of years, India will become home to the largest number of cesarean births in the world.

### Limitations

The precision of the estimates drawn from the last NFHS depends on the sample size and the quality of reporting. The CIs of estimates for smaller states tend to be large. Better estimates of cesarean rates in India will require a systematic monitoring of institutional deliveries by health institutions.

The absence in India of complementary large-scale studies based on the Robson classification^30^ prevents the assessment and analysis of cesarean rates within and between health care facilities. Lack of qualitative studies is a further limitation for understanding the potential factors of overuse of cesarean deliveries in India.

## Conclusions

The proportion of births delivered by cesarean delivery has increased especially fast during the last decade in India, reaching 17.2% (95% CI, 17.1%-17.3%) in 2010 to 2016 according to the NFHS-4 survey. This level is already higher than that observed in some industrialized countries in Europe such as the Netherlands or Finland.^27^ The emerging situation also points to significant regional and sex disparities, with a substantial deficit of cesarean deliveries among underprivileged groups and almost 2 million excess cesarean births every year among more advanced sections of the population.

The need to monitor the further progression of cesarean rates is urgent. The drivers of the current enthusiasm for surgical deliveries in India are not yet well understood. They may include changes in lifestyles, commercial pressure, and cultural factors,^31,32,33^ but this emerging situation calls for further investigation. In urban areas and among the middle class, cesarean rates have already reached levels consistently higher than what is considered medically justified. Effective interventions and policies targeted at women and health care professionals to reduce unnecessary cesarean deliveries^34,35^ will be required in India to avoid growing inequalities in access to cesarean and unnecessary procedures.

## Notes

Supplement. **eMethods 1.** Assessment of the NFHS-4 Figures on Cesarean Births **eMethods 2.** Computation of the Excess, Deficit and Concentration of Cesarean Births **eTable 1.** Population-Based Cesarean Rates in India by State and Union Territory According to 3 Demographic Surveys, 2005-2016 **eTable 2.** Population-Based Cesarean Section Rates in India and Relative Risk by Socioeconomic Quintile and by State and Rural/Urban, National Family Health Survey (NFHS-4), 2010-2016 **eFigure 1.** Population-Based Cesarean Rates in India by State and Union Territory According to the DLHS-4 and the NFHS-4 Surveys, 2011-2016 **eFigure 2.** Lorenz Concentration Curve of Cesarean Deliveries in India, National Family Health Survey (NFHS-4), 2010-2015 **eFigure 3.** Cesarean Rates and Neonatal Mortality Rates in India for States With More Than 20 Million Inhabitants, National Family Health Survey (NFHS-4), 2010-2016
