# Supplementary material for: Trends, Regional Variations, and Socioeconomic Disparities in Cesarean Births in India, 2010-2016
Source: JAMA Netw Open. 2019 Mar 22;2(3):e190526. doi: 10.1001/jamanetworkopen.2019.0526 (PMC6583298; doi:10.1001/jamanetworkopen.2019.0526)
Supplement: Supplement. — eMethods 1. Assessment of the NFHS-4 Figures on Cesarean Births eMethods 2. Computation of the Excess, Deficit and Concentration of Cesarean Births eTable 1. Population-Based Cesarean Rates in India by State and Union Territory According to 3 Demographic Surveys, 2005-2016 eTable 2. Population-Based Cesarean Section Rates in India and Relative Risk by Socioeconomic Quintile and by State and Rural/Urban, National Family Health Survey (NFHS-4), 2010-2016 eFigure 1. Population-Based Cesarean Rates in India by State and Union Territory According to the DLHS-4 and the NFHS-4 Surveys, 2011-2016 eFigure 2. Lorenz Concentration Curve of Cesarean Deliveries in India, National Family Health Survey (NFHS-4), 2010-2015 eFigure 3. Cesarean Rates and Neonatal Mortality Rates in India for States With More Than 20 Million Inhabitants, National Family Health Survey (NFHS-4), 2010-2016 [file jamanetwopen-2-e190526-s001.pdf]

# Supplementary Online Content

Guilmoto CZ, Dumont A. Trends, regional variations, and socioeconomic disparities in cesarean births in India, 2010-2016. *JAMA Netw Open*. 2019;2(3):e190526. doi:10.1001/jamanetworkopen.2019.0526

**eMethods 1.** Assessment of the NFHS-4 Figures on Cesarean Births

**eMethods 2.** Computation of the Excess, Deficit and Concentration of Cesarean Births

**eTable 1.** Population-Based Cesarean Rates in India by State and Union Territory According to 3 Demographic Surveys, 2005-2016

**eTable 2.** Population-Based Cesarean Section Rates in India and Relative Risk by Socioeconomic Quintile and by State and Rural/Urban, National Family Health Survey (NFHS-4), 2010-2016

**eFigure 1.** Population-Based Cesarean Rates in India by State and Union Territory According to the DLHS-4 and the NFHS-4 Surveys, 2011-2016

**eFigure 2.** Lorenz Concentration Curve of Cesarean Deliveries in India, National Family Health Survey (NFHS-4), 2010-2015

**eFigure 3.** Cesarean Rates and Neonatal Mortality Rates in India for States With More Than 20 Million Inhabitants, National Family Health Survey (NFHS-4), 2010-2016

This supplementary material has been provided by the authors to give readers additional information about their work.

© 2019 Guilmoto CZ et al. *JAMA Network Open.*

## eMethods 1. Assessment of the NFHS-4 Figures on Cesarean Births

The preliminary analysis of original NFHS-4 data revealed that very high cesarean birth rates were observed in several states in India. Especially noticeable were the states of Andhra Pradesh and Telangana, where rates reach the unusually high levels of 40% and 58% respectively. The NFHS-4 estimates can be further compared with the state-wise estimates drawn from the previous NFHS round conducted in 2005-06 (eTable 1).

**eTable 1. Population-Based Cesarean Rates in India by State and Union Territory According to 3 Demographic Surveys, 2005-2016**

| Survey | NFHS-4 | NFHS-3 | Difference between NFHS rounds | DLHS-4* |
|---|---|---|---|---|
| Year | 2015-16 | 2005-6 | | 2012-13 |
| Andaman and Nicobar Islands | 19.3% | NA | | 12% |
| Andhra Pradesh | 40.1% | 22.2% | 25.3%** | 34% |
| Arunachal Pradesh | 8.9% | 3.0% | 6.0% | 4% |
| Assam | 13.4% | 5.3% | 8.2% | 10% |
| Bihar | 6.2% | 3.1% | 3.1% | 5% |
| Chandigarh | 22.6% | NA | | 14% |
| Chhattisgarh | 9.9% | 4.1% | 5.7% | 7% |
| Dadra and Nagar Haveli | 16.2% | NA | | NA |
| Daman and Diu | 15.7% | NA | | NA |
| Goa | 31.4% | 25.8% | 5.6% | 31% |
| Gujarat | 18.4% | 8.9% | 9.5% | NA |
| Haryana | 11.7% | 5.3% | 6.4% | 8% |
| Himachal Pradesh | 16.7% | 12.6% | 4.1% | 11% |
| Jammu and Kashmir | 33.1% | 13.5% | 19.6% | NA |
| Jharkhand | 9.9% | 3.9% | 6.0% | 8% |
| Karnataka | 23.6% | 15.5% | 8.0% | 21% |
| Kerala | 35.8% | 30.1% | 5.7% | 37% |
| Lakshadweep | 38.4% | NA | | NA |
| Madhya Pradesh | 8.6% | 3.5% | 5.1% | 6% |
| Maharashtra | 20.1% | 11.6% | 8.5% | 14% |
| Manipur | 21.1% | 9.0% | 12.1% | 11% |
| Meghalaya | 7.6% | 4.1% | 3.5% | 4% |
| Mizoram | 12.7% | 6.2% | 6.5% | 5% |
| Nagaland | 5.8% | 2.0% | 3.8% | 3% |
| Delhi | 26.7% | 13.7% | 13.0% | NA |
| Odisha | 13.8% | 5.1% | 8.7% | 9% |
| Puducherry | 33.6% | NA | | 38% |
| Punjab | 24.6% | 16.6% | 8.0% | 17% |
| Rajasthan | 8.6% | 3.8% | 4.8% | 7% |
| Sikkim | 20.9% | 12.3% | 8.5% | 15% |
| Tamil Nadu | 34.1% | 20.3% | 13.8% | 31% |
| Telangana | 57.7% | NA | | 53% |
| Tripura | 20.5% | 12.9% | 7.6% | 13% |
| Uttar Pradesh | 9.4% | 4.4% | 5.0% | 7% |
| Uttarakhand | 13.1% | 8.1% | 5.0% | 11% |

© 2019 Guilmoto CZ et al. *JAMA Network Open.*

| | | | | |
|---|---|---|---|---|
| **West Bengal** | 23.8% | 10.2% | 13.6% | 23% |
| **India** | 17.2% | 8.5% | 8.7% | |

NFHS: National Family and Health Surveys; DLHS: District-Level Household Survey; NA: data not available for these states

*: AHS data (2011-12) used for Assam, Bihar, Chhattisgarh, Jharkhand, Madhya Pradesh, Odisha, Rajasthan, Uttarkhand, and Uttar Pradesh where DHLS-4 was not conducted.

**: computed over the old Andhra Pradesh state (including Telangana)

Computed from DLHS and NFHS datasets

This comparison points to the very rapid increase observed in the former Andhra Pradesh state, which included then Telangana: the cesarean rate more than doubled in Andhra Pradesh, rising by 25.3 points in a decade. The national in cesarean rates have been significantly slower in India (+8.7%) and in specific regions, notably among other South Indian states such as Kerala (+5.7%), Tamil Nadu (+13.8%) or Karnataka (+8.0%). Thus, in spite of strong congruence between estimates of cesarean rates from the last two NFHS rounds ($r^2$=.81), the proportion of cesarean deliveries seems to have recorded an unusual growth in the states of Andhra Pradesh and Telangana.

In order to test the reliability of the NFHS-4 figure for Andhra Pradesh and Telangana, we conducted two consistency tests at state- and district-level. We used for that the results of the DLHS-4 survey conducted in 2011-12. This is the latest survey-based estimates of cesarean deliveries in India. The DLHS-4 was replaced by the Annual Health Survey (AHS) in nine less advanced poorer states (Assam, Bihar, Chhattisgarh, Jharkhand, Madhya Pradesh, Odisha, Rajasthan, Uttarkhand, and Uttar Pradesh). We use therefore data from the AHS (2011-12) for these states. Cesarean rates from AHS were computed from the rates in government and private institutions mentioned in the respective state AHS publications. In addition, no result has been published by the DLHS-4 for several states and territories including Gujarat. A total of 29 state-wise estimates of cesarean rates are therefore available for comparison between the NFHS-4 and the NFHS-4 and AHS conducted about 2 years earlier (eTable 1).

The values plotted in eFigure 1 demonstrate the very strong correlation ($r^2$=0.94) existing between the two series, in spite of the overall increase in cesarean rates between the two successive surveys. We observe in particular that the NFHS-4 estimates for Telangana and Andhra Pradesh where cesarean rates rose very fast between the last two NFHS rounds are confirmed by the DLHS-4 figures.

© 2019 Guilmoto CZ et al. *JAMA Network Open.*

**eFigure 1. Population-Based Cesarean Rates in India by State and Union Territory According to the DLHS-4 and the NFHS-4 Surveys, 2011-2016**

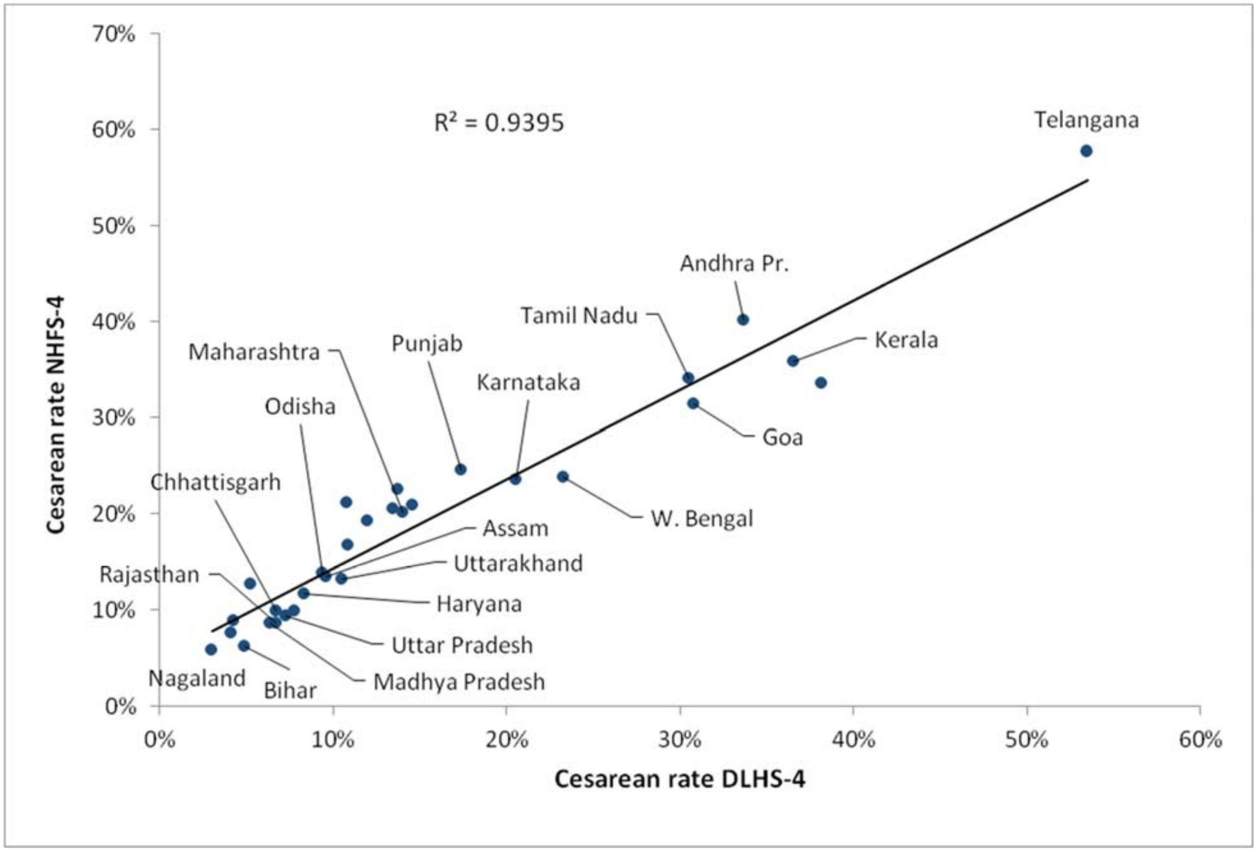


We conducted a further district-level analysis for the former state of Andhra Pradesh (Andhra Pradesh and Telangana). We plotted again the DHLS-4 estimates against the NFHS-4 estimates for the 23 districts that constitute today Andhra Pradesh and Telangana. The correlation coefficient ($r^2$=0.65) between the two series is high and significant at 1%, while lower than the coefficient measured at state-level because of the higher variability of district-level estimates. This strong correlation confirms the consistency of NFHS-4 figures in the two states where cesarean rates recorded the fastest growth during the last ten years.

.

© 2019 Guilmoto CZ et al. *JAMA Network Open.*

**eTable 2. Population-Based Cesarean Section Rates in India and Relative Risk by Socioeconomic Quintile and by State and Rural/Urban, National Family Health Survey (NFHS-4), 2010-2016**

| State | Poorest | | Poor | | Middle | | Richer | | Richest | | Total | | Relative risk* |
|---|---|---|---|---|---|---|---|---|---|---|---|---|---|
| Unit | % (95% CI) | Births | % (95% CI) | Births | % (95% CI) | Births | % (95% CI) | Births | % (95% CI) | Births | % (95% CI) | Births | Ratio (95% CI) |
| Andhra Pradesh | 19.6 (13.2-26) | 149 | 26.9 (23.2-30.6) | 553 | 37.5 (34.6-40.3) | 1118 | 46.7 (43.3-50) | 865 | 57.1 (52.5-61.7) | 443 | 40.1 (38.4-41.8) | 3128 | 2.9 (2.1-3.9) |
| Arunachal Pradesh | 1.4 (0.7-2) | 1239 | 3 (2.1-3.9) | 1339 | 8 (6.5-9.5) | 1225 | 20.2 (17.5-22.9) | 846 | 28.6 (23.6-33.6) | 317 | 8.9 (8.1-9.7) | 4966 | 21.1 (13-32.1) |
| Assam | 3.3 (2.7-4) | 3256 | 8.1 (7.2-8.9) | 4016 | 19.7 (17.9-21.6) | 1782 | 39.2 (36.1-42.4) | 924 | 60.1 (54.8-65.3) | 331 | 13.4 (12.7-14.1) | 10309 | 18 (14.9-21.7) |
| Bihar | 3.4 (3.1-3.7) | 14223 | 5.9 (5.3-6.5) | 6145 | 10.2 (9.1-11.3) | 2910 | 19.5 (17.6-21.4) | 1668 | 28.6 (24.6-32.6) | 491 | 6.2 (5.9-6.5) | 25437 | 8.5 (7.7-9.9) |
| Chhattisgarh | 3.3 (2.7-3.9) | 3588 | 6.1 (5.1-7.2) | 2151 | 10.5 (8.9-12.1) | 1448 | 15.7 (13.5-18) | 1047 | 29.8 (27-32.6) | 1049 | 9.9 (9.3-10.5) | 9283 | 9.1 (7.5-10.9) |
| Gujarat | 5.8 (4.4-7.1) | 1140 | 5.9 (4.8-6.9) | 1804 | 11.5 (10.1-13) | 1852 | 25.7 (23.5-27.9) | 1574 | 35.1 (32.6-37.7) | 1360 | 18.4 (17.5-19.3) | 7730 | 6.1 (4.8-7.6) |
| Haryana | 4.6 (2-7.1) | 251 | 5.1 (3.5-6.6) | 770 | 6.5 (5.2-7.8) | 1476 | 9.8 (8.5-11.1) | 2115 | 17.3 (16-18.6) | 3270 | 11.7 (11-12.4) | 7882 | 3.8 (2.1-6) |
| Himachal Pradesh | 2.4 (-1.7-6.5) | 54 | 4.6 (2.4-6.9) | 340 | 10 (7.8-12.3) | 703 | 15.2 (13-17.3) | 1052 | 28.7 (25.5-31.9) | 780 | 16.7 (15.3-18) | 2929 | 11.8 (2.2-32.6) |
| Jammu and Kashmir | 8.6 (6.9-10.3) | 1029 | 17.6 (16-19.1) | 2215 | 33 (31-35) | 2115 | 44.7 (42.2-47.2) | 1573 | 50 (47.3-52.7) | 1313 | 33.1 (32.1-34.1) | 8245 | 5.8 (4.8-7) |
| Jharkhand | 3.8 (3.3-4.3) | 6346 | 8.6 (7.6-9.7) | 2649 | 15.7 (13.9-17.4) | 1592 | 22.1 (19.6-24.7) | 988 | 39.6 (35.7-43.4) | 629 | 9.9 (9.3-10.4) | 12204 | 10.4 (9.1-12) |
| Karnataka | 12.7 (10-15.3) | 595 | 14.6 (13-16.2) | 1861 | 21.6 (20-23.2) | 2457 | 26.3 (24.4-28.3) | 2000 | 37.8 (34.6-41) | 876 | 23.6 (22.6-24.5) | 7789 | 3 (2.4-3.7) |
| Kerala | 41.6 (16.7-66.5) | 15 | 26.5 (15.9-37.2) | 66 | 36.5 (31.5-41.6) | 353 | 36.7 (33.7-39.7) | 973 | 35.1 (32.3-38) | 1055 | 35.8 (33.9-37.7) | 2462 | 0.8 (0.5-1.2) |
| Madhya Pradesh | 2.3 (2-2.6) | 8878 | 4.4 (3.9-4.9) | 5896 | 8.1 (7.2-9) | 3762 | 15.1 (13.9-16.4) | 3268 | 28.9 (27.2-30.5) | 2807 | 8.6 (8.3-9) | 24611 | 12.5 (10.9-14.4) |
| Maharashtra | 5.3 (4-6.6) | 1171 | 9.6 (8.3-11) | 1931 | 16.6 (15.1-18.1) | 2400 | 23.3 (21.6-25.1) | 2268 | 36.6 (34.3-39) | 1631 | 20.1 (19.3-20.9) | 9401 | 6.9 (5.4-8.6) |
| Manipur | 3.9 (2.6-5.3) | 788 | 10.4 (9-11.8) | 1917 | 20.8 (18.8-22.8) | 1598 | 34.5 (31.5-37.6) | 928 | 51.4 (46.5-56.3) | 405 | 21.1 (20.1-22.2) | 5636 | 13.1 (9.2-17.8) |
| Meghalaya | 2.6 (1.5-3.8) | 731 | 3.2 (2.4-4.1) | 1768 | 9.8 (8.3-11.4) | 1353 | 20.8 (16.9-24.6) | 425 | 29.4 (21.7-37.2) | 132 | 7.6 (6.8-8.4) | 4409 | 11.2 (7.1-17.1) |
| Mizoram | 1.5 (0.3-2.7) | 389 | 4.6 (3.2-6.1) | 798 | 6 (4.7-7.2) | 1295 | 13.2 (11.5-15) | 1439 | 24.5 (21.8-27.2) | 984 | 12.7 (11.7-13.6) | 4905 | 16.3 (7.3-29.7) |
| Nagaland | 0.4 (0-0.8) | 885 | 2 (1.4-2.7) | 1852 | 6.3 (4.8-7.8) | 1045 | 13.3 (10.5-16.2) | 546 | 23.5 (18.5-28.4) | 279 | 5.8 (5.1-6.5) | 4607 | 63.3 (21.4-136.6) |
| Odisha | 6.2 (5.5-6.9) | 4564 | 11.4 (10.2-12.5) | 2872 | 16.9 (15.3-18.6) | 1992 | 30.7 (28-33.5) | 1110 | 40.6 (36.5-44.6) | 568 | 13.8 (13.2-14.4) | 11106 | 6.5 (5.8-7.5) |
| Punjab | 11.3 (2.7-19.9) | 52 | 8.4 (4.8-11.9) | 232 | 15.7 (13.1-18.3) | 752 | 17.6 (15.5-19.6) | 1278 | 31 (29.3-32.7) | 2902 | 24.6 (23.4-25.7) | 5216 | 2.7 (1.3-4.7) |
| Rajasthan | 2.3 (1.8-2.8) | 3640 | 4.4 (3.8-5) | 4185 | 7.7 (6.9-8.6) | 3539 | 11 (9.9-12.2) | 2869 | 22.2 (20.6-23.8) | 2599 | 8.6 (8.2-9) | 16832 | 9.8 (7.9-12) |
| Tamil Nadu | 30.7 (25.2-36.1) | 275 | 25.4 (23-27.8) | 1271 | 32.1 (30.3-34) | 2524 | 35.7 (33.9-37.6) | 2561 | 41.5 (38.8-44.2) | 1291 | 34.1 (33-35.1) | 7922 | 1.4 (1.1-1.6) |
| Uttar Pradesh | 3 (2.7-3.3) | 12937 | 5.3 (4.9-5.8) | 10151 | 10 (9.4-10.7) | 7534 | 14.1 (13.2-15) | 5970 | 29.2 (27.9-30.4) | 5159 | 9.4 (9.1-9.7) | 41751 | 9.7 (8.8-10.8) |
| Uttarakhand | 3 (1.2-4.7) | 364 | 3.3 (2.3-4.3) | 1275 | 7.1 (5.9-8.3) | 1647 | 16.2 (14.2-18.2) | 1308 | 27 (24.6-29.5) | 1231 | 13.1 (12.2-14) | 5825 | 9.1 (5-14.6) |
| West Bengal | 8.6 (7.2-10) | 1508 | 14.6 (13-16.2) | 1791 | 28.5 (25.8-31.2) | 1063 | 45.1 (41.4-48.8) | 690 | 74.9 (69.7-80) | 276 | 23.8 (22.7-25) | 5328 | 8.7 (7.3-10.2) |
| Telangana | 37.9 (31.1-44.8) | 192 | 41.3 (37-45.5) | 511 | 54.2 (50.4-58) | 668 | 64.3 (60.7-67.9) | 671 | 71.4 (66.9-75.9) | 385 | 57.7 (55.8-59.7) | 2427 | 1.9 (1.6-2.2) |
| Rural India | 4.3 (4.2-4.5) | 65664 | 9.5 (9.3-9.8) | 55036 | 18.4 (18.1-18.8) | 40028 | 25.2 (24.7-25.7) | 24371 | 29.1 (28.3-29.9) | 13149 | 12.8 (12.7-13) | 198248 | 6.7 (6.4-7) |
| Urban India | 5.8 (5-6.6) | 3032 | 10.7 (10-11.5) | 6400 | 20.7 (20-21.4) | 11734 | 28.5 (27.8-29.1) | 18575 | 38.8 (38.2-39.5) | 21638 | 28.2 (27.9-28.6) | 61379 | 6.7 (5.8-7.7) |
| India | 4.4 (4.3-4.6) | 68696 | 9.7 (9.4-9.9) | 61436 | 19 (18.7-19.4) | 51762 | 26.8 (26.4-27.2) | 42946 | 35.9 (35.4-36.4) | 34787 | 17.2 (17-17.3) | 259627 | 8.1 (7.8-8.4) |

Based on cesarean rates computed by socioeconomic quintile for the states with more than 2000 sample births. To calculate relative inequality, we divided the CS rate in the richest quintile by the CS rate in the poorest quintile. The same computations were replicated separately for rural and urban areas.

* Relative risk (inter-quintile ratio) computed as the ratio of rates in the richest and poorest quintiles

- Values are percentages of cesarean births among all births.
- States with fewer than 2000 samples births omitted

© 2019 Guilmoto CZ et al. *JAMA Network Open.*

## eMethods 2. Computation of the Excess, Deficit and Concentration of Cesarean Births

Following WHO latest indications, we use the values of 10 and 15% as lower and upper limits of cesarean births. We consider proportions below or above this range to represent respectively a deficit or an excess of cesarean deliveries.

The easiest way to compute any excess cesarean births would be to use the all-India rate of 17.2% and to estimate the excess above the 15% threshold as 2.1% of deliveries. This, however, ignores the considerable variations observed across regions or social groups that are documented in this paper (Table 2, eTable 2). Bihar for instance recorded a proportion of 6.2%, pointing to the presence of a significant deficit of 3.8 in a major state of India. In contrast, Kerala recorded a cesarean rate of 35.9%, pointing to a potential excess by 20.9 percentage points in this state. Cesarean rates similarly vary along a broad range according to wealth quintile or place of delivery. We also rejected the place of delivery as a criterion since the place of delivery may be as much a cause as a consequence of the delivery methods: in case of pregnancy complications, poor women may for instance be referred to a public hospital instead of delivering at home, while middle-class women in the same situation may opt for private facilities instead.

To estimate the excess or deficit of cesarean deliveries, we therefore decided to divide the NFHS-4 sample into more homogenous subgroups, using states, districts, fertility levels, and socioeconomic quintiles for that purpose. For each subgroup, we counted excess or deficit cesarean births by using the 10-15% threshold. A purely regional division (by states or districts) was abandoned as it proved unable to capture the socioeconomic variations within regional units. It may, for instance, be observed that the richest quintile in Bihar—a state with low cesarean rates—had cesarean rates close to 30%, i.e. distinctly above the 15% threshold. Inversely, the poorest quintile in Gujarat—a state with high cesarean rates—recorded a cesarean rate of 6% below the 10% threshold. The division by fertility levels also proved insufficient. The best disaggregation was obtained by dividing the NFHS-4 sample by states and socioeconomic quintiles. The resulting 180 groups (36 states x 5 quintiles) had an average 1442 births. These subgroups yielded a detailed partition of India into homogenous regional and economic subpopulations. We then computed the deficit or the excess of cesarean births in each of these 180 subgroups by using the 10-15% range.

A majority of these subgroups (97) had cesarean rates above 15%, half of them with rates greater than 30%. When cumulated over these subgroups, the excess cesarean deliveries amount to an overall excess of cesarean deliveries of 7.0% of all deliveries. In contrast, a substantial number (65) of subgroups recorded on the contrary rates below 10% and half of them less than 5%. The overall shortfall in cesarean births among them affects 2.2% of deliveries. When we restricted our computation to cesarean rates significantly different from the 10-15% range in each subgroup, we obtained an identical number of deficit and excess cesarean births. Using the estimated number of annual births in India during this period derived from United Nations

© 2019 Guilmoto CZ et al. *JAMA Network Open.*

estimates and the proportion of excess and deficit cesarean deliveries, we further computed the total deficit of cesarean births at 0.5 million per year and the total excess at 1.8 million per year in 2010-16.

The division of the Indian sample into 180 subgroups was also used to draw the Lorenz concentration curve in which subgroups are ranked by increasing cesarean rates (eFigure 2). The observed distribution of cesarean deliveries (in red) can be related to the equal distribution (in blue). The curve illustrates the gap between Indian subpopulations: the less privileged 40% of births account only for 10% of all cesarean deliveries while less than 20% of the births among better-off populations account for half of the cesarean deliveries. The Gini coefficient of inequality presented in this paper was computed as the relative difference between the observed and equal distributions derived from the Lorenz curve.

© 2019 Guilmoto CZ et al. *JAMA Network Open.*

**eFigure 2. Lorenz Concentration Curve of Cesarean Deliveries in India, National Family Health Survey (NFHS-4), 2010-2015**

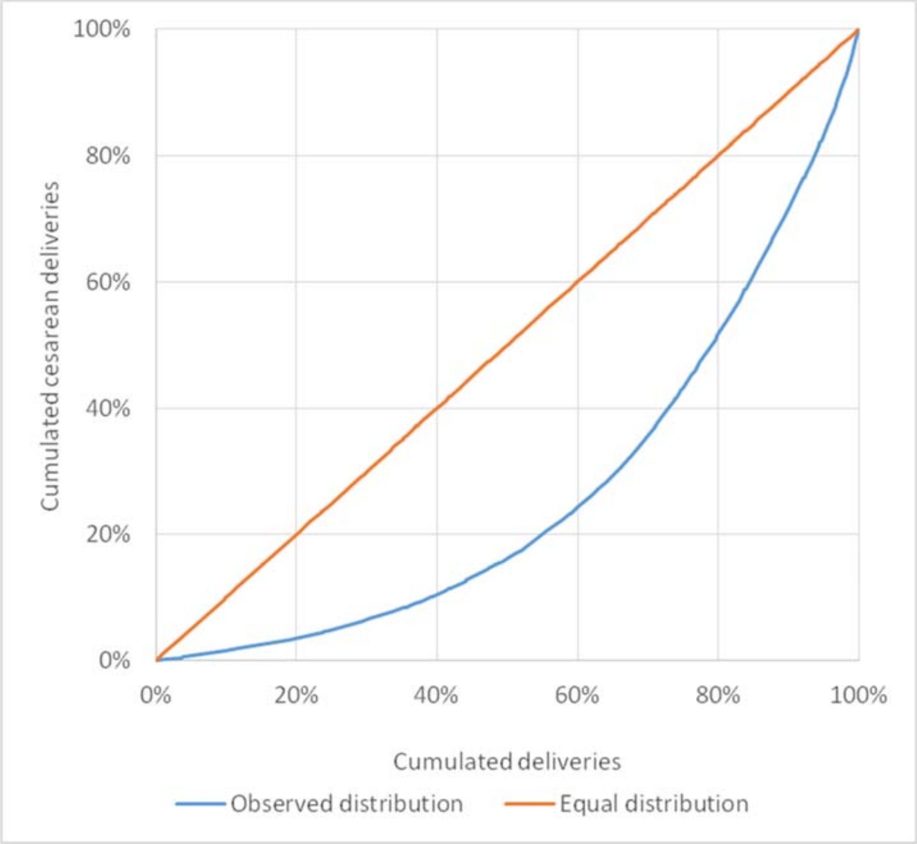


© 2019 Guilmoto CZ et al. *JAMA Network Open.*

**eFigure 3. Cesarean Rates and Neonatal Mortality Rates in India for States With More Than 20 Million Inhabitants, National Family Health Survey (NFHS-4), 2010-2016**

Sources: NFHS-4, 2010-2016

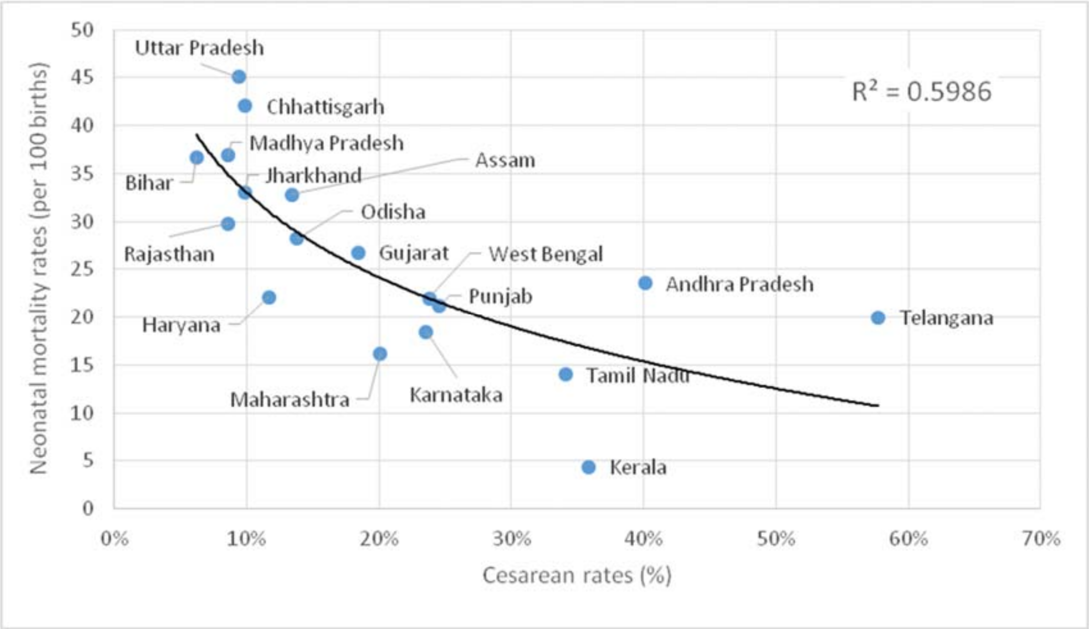


© 2019 Guilmoto CZ et al. *JAMA Network Open.*
